# In Vitro Lymphocyte Functions in Undernourished Children With Sickle Cell Anemia

**DOI:** 10.31486/toj.19.0024

**Published:** 2020

**Authors:** Solo R. Kuvibidila, Renée Gardner, Maria C. Velez, Lolie Yu, Rajasekharan P. Warrier

**Affiliations:** ^1^ Department of Pediatrics, Division of Hematology/Oncology, Louisiana State University Health Sciences Center, New Orleans, LA; ^2^ Department of Pediatrics, Division of Hematology/Oncology, Ochsner Children's Hospital, New Orleans, LA; ^3^ The University of Queensland Faculty of Medicine, Ochsner Clinical School, New Orleans, LA

**Keywords:** *Albumin*, *anemia–sickle cell*, *anthropometry*, *ceruloplasmin*, *C-reactive protein*, *interleukin-2*, *lymphocyte proliferation*, *prealbumin*, *retinol-binding protein*, *undernutrition*

## Abstract

**Background:** Children with sickle cell disease (SCD) often suffer from growth deficits and impaired immunity. However, the association between mild to moderate malnutrition and in vitro lymphocyte function has not been well studied. The goal of this study was to investigate the effects of undernutrition on lymphocyte functions in children with SCD.

**Methods:** Weight; height; plasma concentrations of albumin (Alb), prealbumin (PA), transferrin (Tf), retinol-binding protein (RBP), α_1_-acid glycoprotein (AGP), C-reactive protein (CRP), and ceruloplasmin (Cp); and lymphocyte proliferation and interleukin (IL)-2 in phytohemagglutinin-treated blood lymphocytes were measured in 90 children with SCD (59 SS, 4 Sβ°, 27 SC hemoglobin genotypes).

**Results:** The mean age of the children included in the analysis was 7.65 years. Four of the 90 children had weight and height below the fifth percentile. A higher percentage of children with HbSS/HbSβ° (61.4%) than of those with HbSC (44%) had ≥2 plasma protein concentrations below normal (Alb <35 g/L, PA <160 mg/L, Tf <2.0 g/L, and RBP ≤20 mg/L). Mean anthropometric measurements*,* hemoglobin, and hematocrit were lower in children with HbSS/HbSβ° than in those with HbSC (*P*<0.05). Lymphocyte proliferation was reduced by 20% to 27% in children with HbSS/HbSβ° with undernutrition plus inflammation (AGP >1 g/L, CRP >5 mg/L, Cp >600 mg/L) compared to children with neither. Regardless of inflammatory status, lymphocyte proliferation was reduced by 29% to 49% in children with HbSS/HbSβ° and undernutrition defined by PA or Alb plus RBP (*P*<0.05) compared to those with RBP within normal range. Neither undernutrition nor inflammation reduced lymphocyte proliferation in children with HbSC. Mean IL-2 activity was reduced by undernutrition, defined as PA <160 mg/L, in both groups. PA, RBP, and hemoglobin concentrations positively correlated with in vitro lymphocyte functions (*P*<0.05).

**Conclusion:** Undernutrition altered in vitro lymphocyte function in children with the HbSS/HbSβ° genotypes. Dietary supplements may improve the altered functions in these children.

## INTRODUCTION

Severe protein-energy malnutrition (PEM) and/or micronutrient deficiencies in humans and laboratory animals impair innate and adaptive immunity.^1-3^ However, the effects of mild to moderate PEM on immune function is less clear. We do know that mild to moderate PEM in humans impairs some (eg, delayed type hypersensitivity) but not other (eg, antibody responses to vaccination) immune functions.^4^ Lymphocyte proliferation, one of the tests used to assess in vitro lymphocyte function, is usually reduced by severe PEM.^1,3^ The mechanisms involved may include changes in lymphocyte subsets and reduced secretion or gene expression of cytokines and various molecules (eg, interleukin [IL]-2, IL-4, IL-17, cyclins) that regulate cell proliferation.^1^

Young children with sickle cell disease (SCD), especially those with the hemoglobin (Hb)-SS genotype, often have growth deficits and impaired immunity.^4-8^ The mechanisms of growth deficits are multifactorial and involve inadequate food intake (eg, during episodes of pain crisis and/or infection), increased resting energy expenditure because of increased red blood cell production, and increased nutrient loss resulting from red blood cell destruction.^9-10^ Reduced nutrient absorption—specifically vitamins and minerals—during infection or because of chronic inflammation is another mechanism that may lead to nutrient insufficiency.^10-13^ Providing dietary supplementation with zinc and/or other nutrients to children with growth deficits has resulted in improved growth measurements in studies with small cohorts.^14-17^

Possible mechanisms of impaired immunity include nutrient deficiencies, iron overload because of blood transfusion, alloimmunization, and splenic dysfunction.^14^ Specific changes in adaptive and innate immunity include the reduced proportion of circulating CD4+ and CD8+ T cells and a shift from TH1 (interferon gamma and IL-2) to TH2 (IL-6 and IL-4) cytokine response.^10^ Elevated numbers of nonfunctional neutrophils, defective macrophages, natural killer cells, and T regulatory cells have also been observed.^11^ Increased blood levels of proinflammatory cytokines, including tumor necrosis factor alpha (TNF-α) and acute phase proteins (eg, C-reactive protein [CRP]), have been reported as well.^12-18^ A few studies, although conducted on a limited number of children, have shown that dietary supplements (eg, zinc) improved in vitro immune function and reduced infection rates.^14,16,17^

We have observed that lymphocyte proliferation and IL-2 secretion tend to be lower in children with SCD who have suboptimal vitamin A status vs those with adequate plasma retinol levels.^19^ In a previous small study, we reported that nearly 53% of the children with SCD had plasma levels of retinol-binding protein (RBP) <30 mg/L, but no child had albumin (Alb) levels <35 g/L.^20^ The study suggested the presence of mild to moderate PEM in our SCD patient population*.* The effects of mild to moderate PEM on in vitro lymphocyte function in patients with SCD have not been well studied*.*

We designed this study to investigate (1) the effect of mild to moderate PEM on in vitro immune responses of peripheral blood mononuclear cells in children with SCD and (2) whether Hb genotype and inflammation are confounding factors in impaired lymphocyte function.

## METHODS

### Study Population and Design

The study was approved by the institutional review boards of Louisiana State University Health Sciences Center and the Children's Hospital of New Orleans. Patients included in the analysis presented in this paper were enrolled in a prospective study in which we sought to investigate the associations among nutritional status, in vitro immune functions, inflammatory cytokines, and certain complications (including pain crisis episodes, infection, blood transfusion requirements, and hospitalizations) often observed in children with SCD. Our data on inflammatory cytokines (TNF-α), the association between vascular cell adhesion molecule-1 and zinc levels, lymphocyte proliferation as a function of plasma retinol concentration, and clinical status have been previously published.^18,19,21^

Inclusion criteria were boys and girls with HbSS, HbSC, and/or HbSβ^thalassemia^ (HbSβ^thal-0^) genotypes who were 0.5 to 18 years old. Exclusion criteria were children who (1) were on hydroxyurea at the time of recruitment, (2) had received a bone marrow transplant, (3) had had a splenectomy, and (4) were hospitalized at the time of blood drawing. Ninety children met the inclusion criteria.

Because only 4 children had the HbSβ° genotype and because the disease in children with this genotype is usually as severe as that observed in children with HbSS, we combined the 2 subgroups for analysis.^22^

### Measurement of Indicators of Nutritional Status and Inflammation

Blood samples were collected in heparinized vacutainers in children under stable conditions. After removing 200 μL of blood for the measurement of Hb, hematocrit, and white blood cell count by standard techniques, the remaining blood samples were centrifuged at 400 × g for 10 minutes. Plasma was aspirated and immediately frozen at –70°C until used for various measurements.

Biochemical indicators of nutritional status—Alb, prealbumin (PA), transferrin (Tf), and RBP—were measured in plasma by radial immunodiffusion. The cutoff points to define undernutrition were Alb <35 g/L, PA <160 mg/L, Tf <2.0 g/L, and RBP ≤20 mg/L.^23,24^ Growth deficits were present when either weight or height was below the fifth percentile. Regardless of the weight and height percentile, children with at least 2 plasma proteins below the proposed cutoff points were considered to have mild to moderate PEM.

Inflammation was assessed through the measurement of CRP, α_1_-acid glycoprotein (AGP), and ceruloplasmin (Cp) in plasma by radial immunodiffusion. Inflammation was defined based on the most recent cutoff points proposed by the World Health Organization: AGP >1 g/L, CRP >5 mg/L, and Cp >600 mg/L.^25,26^

### Lymphocyte Proliferation

Details for the isolation of mononuclear cells and culture conditions for lymphocyte proliferation have been previously described.^19,27^ The culture medium contained RPMI-1640, 10% heat-inactivated human AB serum, 5 × 10^4^ units penicillin, 50 mg streptomycin, 1 mmol sodium pyruvate, 0.1 mmol nonessential amino acids, 2 mmol L-glutamine, and 50 μmol β-mercaptoethanol (per 1,000 mL). Lymphocyte proliferation (of triplicate cultures containing 2 × 10^5^ viable cells/200 μL medium) was assessed by the rate of ^3^H-thymidine incorporated into DNA.^18^ Results are expressed as stimulation indexes that are the ratios of counts per minute (CPM) in the presence of 2.5 to 20 μg/mL phytohemagglutinin (PHA) over baseline CPM (obtained in cells incubated without PHA).

### Interleukin-2 Study

Macrocultures (2 × 10^6^ viable cells/mL) containing 0 to 20 μg PHA were incubated at 37°C and 5% CO_2_ in a humidified atmosphere.^19,28^ After 48 hours, cultures were centrifuged, and the supernatant was collected and immediately frozen at –70°C. The IL-2 activity in the supernatant was estimated by the growth rate of IL-2–dependent CTLL-2 cells assessed by ^3^H-thymidine uptake.^27^ In the absence of IL-2, CTLL-2 cells (which are derived from murine cytotoxic T cells) do not undergo cell multiplication*.*^28^

### Statistical Analysis

Means ± standard error of the mean (SEM), analysis of variance, Pearson correlation coefficients, and chi-square test were performed by Microstat Program (Microsoft Inc.).^29^

Lymphocyte proliferative responses to PHA and IL-2 concentrations were analyzed as a function of Hb genotypes, undernutrition, and inflammation. Multiple regression analysis was computed with IL-2 and/or lymphocyte proliferation as dependent variables and health status (weight, height, acute phase proteins, transport proteins, and hematologic measurements) as independent variables. The level of significance was set at *P*<0.05. *P* values between 0.05 and 0.07 were considered to indicate a trend of difference.

## RESULTS

### Assessment of Nutritional Status

Demographic data of the patients are summarized in Table 1. The mean ± SEM age of the 90 patients (50 boys and 40 girls) was 7.65 ± 0.45 years*.* The percent of children by Hb genotype was 65.6% HbSS, 30% HbSC, and 4.4% HbSβ^o^. Children with the HbSC genotype were younger than those with the HbSS/HbSβ^o^ genotypes (*P*<0.05).

**Table 1.** Demographic Variables and Mean Indicators of Nutritional Status, Hematologic Measurements, and Acute Phase Proteins as a Function of Hemoglobin Genotype

	HbSS/HbSβ° Genotypes	HbSC Genotype
	Subgroup	Subgroup
Variable/Indicator	n=63	n=27
Boys: Girls	34:29	16:11
Age, years	8.28 ± 0.53^a^	6.46 ± 0.86^b^
Weight percentile	33.75 ± 3.70^b^	63.41 ± 6.87^a^
Weight Z-score	–0.52 ± 0.14^b^	0.45 ± 0.10^a^
Height percentile	38.05 ± 4.17^b^	68.73 ± 6.75^a^
Height Z-score	–0.37 ± 0.15^b^	0.92 ± 0.24^a^
White blood cells × 10^6^/mL	12.38 ± 0.68^a^	9.49 ± 0.78^b^
Hemoglobin, g/dL	8.27 ± 0.18^b^	11.11 ± 0.26^a^
Hematocrit, %	26.22 ± 0.60^b^	32.39 ± 0.49^a^
Albumin, g/L	41.54 ± 0.98	38.99 ± 1.45
Prealbumin, mg/L	133.73 ± 7.16^b^	160.72 ± 12.65^a^
Transferrin, g/L	2.36 ± 0.07	2.52 ± 0.13
Retinol binding protein, mg/L	18.70 ± 0.95^b^	25.82 ± 1.93^a^
α_1-_acid-glycoprotein, g/L	0.66 ± 0.03	0.73 ± 0.04
C-reactive protein, mg/L	5.87 ± 1.52	2.35 ± 1.01
Ceruloplasmin, mg/L	495.50 ± 18.35	478.00 ± 29.00

Notes: Data are presented as means ± standard error of the mean. For each measurement, means of both groups with different superscript letters are statistically different: a>b; *P*<0.05.

Children in the HbSS/HbSβ^o^ subgroup had significantly lower means for weight and height percentiles and for weight and height Z-scores than those in the HbSC subgroup (*P*<0.05). At the time of blood drawing, 4 children (3 with HbSS and 1 with HbSβ°) had weight and height less than the fifth percentile.

The white blood cell count mean was higher, and the means of Hb and hematocrit were lower in the subgroup of children with HbSS/HbSβ° vs the subgroup of children with HbSC (*P*<0.05).

The mean concentrations of Alb were within the normal range for both subgroups of children (Table 1). While the mean concentration of PA was within the normal range in children with the HbSC genotype, the PA mean was below normal in the children with the HbSS/HbSβ° genotypes. The mean concentration of RBP was also below normal in children with the HbSS/HbSβ° genotypes. Children with the HbSS/HbSβ° genotypes had lower mean concentrations of PA, Tf, and RBP than the children with the HbSC genotype (*P*<0.05). A slightly and nonsignificantly higher percentage of children with the HbSS/HbSβ° genotypes (61.4%) vs children with the HbSC genotype (44%) had 2 to 4 plasma proteins in the range suggestive of undernutrition (χ^2^=1.5, *P*=0.22).

We observed no significant difference in the mean concentrations of AGP and Cp among both subgroups of children (Table 1). Although the mean CRP concentration in children with HbSS/HbSβ° genotypes was approximately 2.5 times higher than that for children with the HbSC genotype, the difference was not significant.

The percentages of children with inflammation, defined by any of the 3 acute phase proteins, were approximately 44% and 45% for children with the HbSS/HbSβ° and HbSC genotypes, respectively.

### Lymphocyte Proliferation as a Function of Hemoglobin Genotype and Biochemical Indicators of Nutritional Status

**Figure 1.**
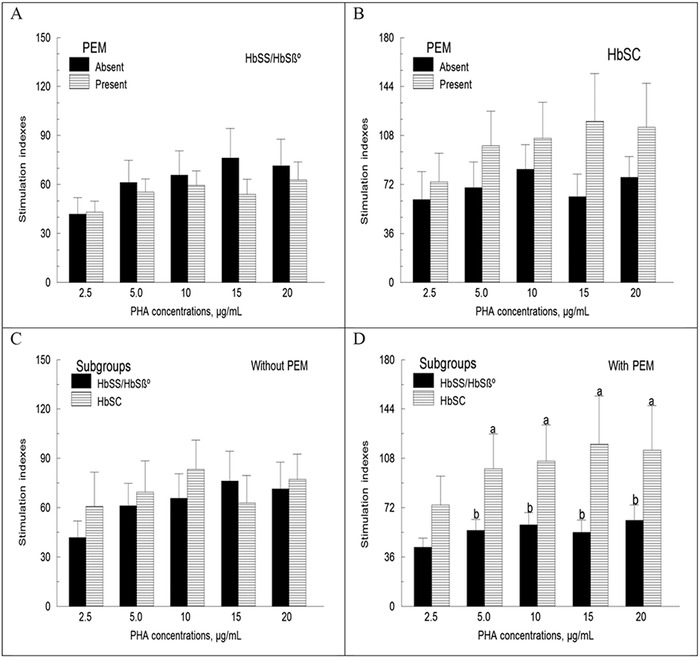
**Lymphocyte proliferation as a function of mild to moderate protein-energy malnutrition (PEM) and hemoglobin (Hb) genotype expressed as stimulation indexes. Mononuclear cells were incubated with and without phytohemagglutinin (PHA) for 48 hours before being pulsed with ^3^H-thymidine for 24 hours. Results are expressed as stimulation indexes or the ratio of the amount of radioactivity incorporated into DNA by cells incubated with PHA over baseline. Sample sizes for PEM absent vs PEM present are as follows: HbSS/HbSβ°, 21 vs 34 and HbSC, 12 vs 15. Values are means ± standard error of the mean. In** **Figure 1****D, the means of patients in the HbSC subgroup are higher than those of patients in the HbSS/HbSβ° subgroup (a>b; *P*<0.05).**

In the subgroup of children with the HbSS/HbSβ° genotypes, mild to moderate PEM was associated with a nonsignificant decrease (9.6% to 29%, mean 15%) in mean lymphocyte proliferative responses to PHA concentrations (Figure 1A). In children with the HbSC genotype, the opposite was observed: mild to moderate PEM was associated with a 22% to 88% increase in mean stimulation indexes compared to those without PEM (Figure 1B). In children without PEM, we found no significant difference in mean lymphocyte proliferative responses to PHA concentrations between the Hb genotype subgroups (Figure 1C). In children with PEM, lymphocyte proliferation was lower in the subgroup of children with the HbSS/HbSβ° genotypes vs those with the HbSC genotype (*P*<0.05) (Figure 1D).

We also compared mean stimulation indexes among children with the HbSS/HbSβ° genotypes as a function of RBP combined with either PA, Alb, or Tf. Four subgroups were defined: (1) normal range concentrations of RBP and the other protein, (2) normal RBP and low concentration of the other protein, (3) below normal RBP and normal concentration of the other protein, and (4) below normal concentrations of RBP and the other protein.

**Figure 2.**
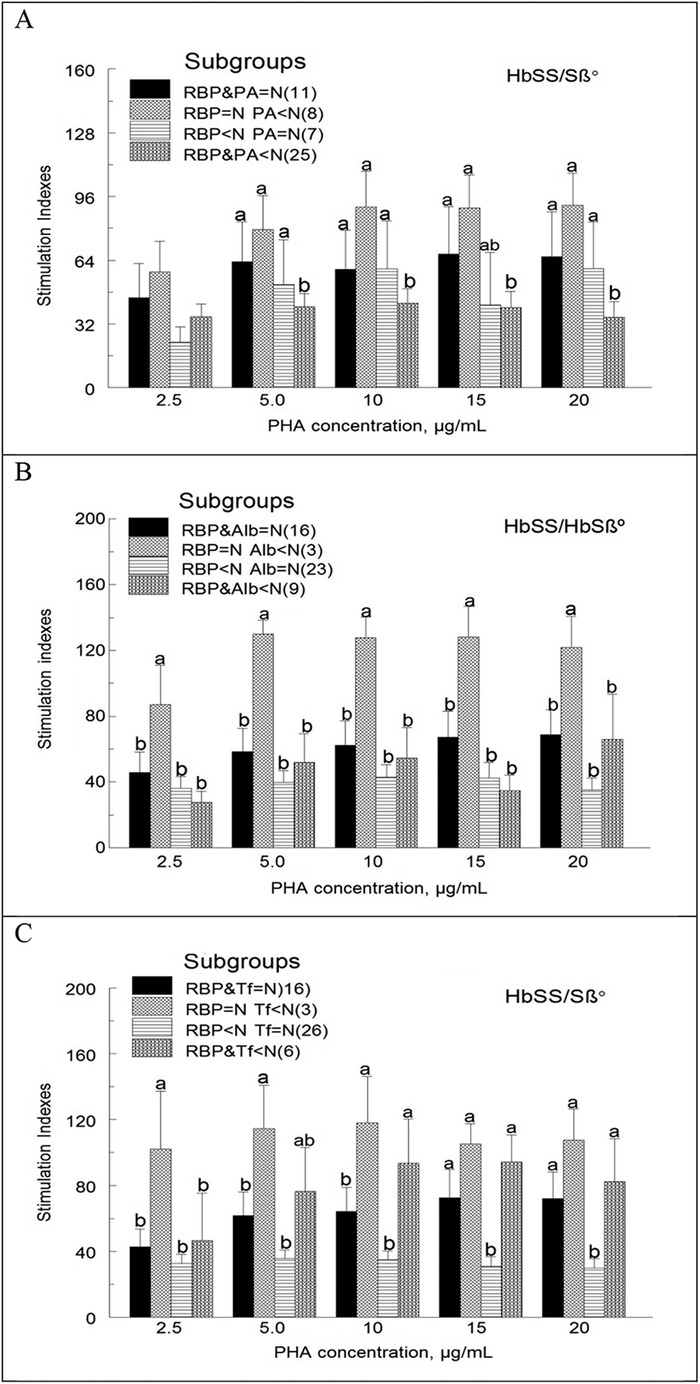
**The effects of retinol binding protein (RBP) in combination with other transport proteins on lymphocyte proliferation in the subgroup of children with the HbSS or HbSβ° genotype. Mononuclear cells were incubated with and without phytohemagglutinin (PHA) before being pulsed with ^3^H-thymidine as explained in the legend for Figure 1. The 4 subgroups in view A are as follows: normal RBP and prealbumin (PA); normal RBP, below normal PA; below normal RBP, normal PA; below normal RBP and PA. In views B and C, PA is replaced by albumin (Alb) and transferrin (Tf), respectively. For each subgroup, numbers in parentheses are the sample sizes. Values are means ± standard error of the mean. For each PHA concentration, mean stimulation indexes (depicted as bars) among the 4 subgroups denoted with the letter a are higher than those denoted with the letter b (a>b; *P*<0.05).**

Regardless of the PA and Alb concentrations (normal or below normal range), mean lymphocyte proliferative responses of cells obtained from children with the HbSS/HbSβ° genotypes and RBP below normal (subgroups 3 and 4) tended to be lower than those of children with RBP within the normal range (subgroups 1 and 2) (Figures 2A and 2B). Mean lymphocyte proliferation also tended to be lower in children with the HbSS/HbSβ° genotypes, RBP below normal, and Tf within or below normal range (subgroups 3 and 4) vs with those with normal RBP concentrations and Tf below normal range (subgroup 2) (Figure 2C). Differences were statistically significant (*P*<0.05) among the 4 subgroups for some but not all PHA concentrations (Figure 2).

We did not analyze data as a function of RBP in combination with the other 3 transport proteins for children with the HbSC genotype because too few children met the criteria for the 4 subgroups defined above.

### Lymphocyte Proliferation as a Function of Inflammation

**Figure 3.**
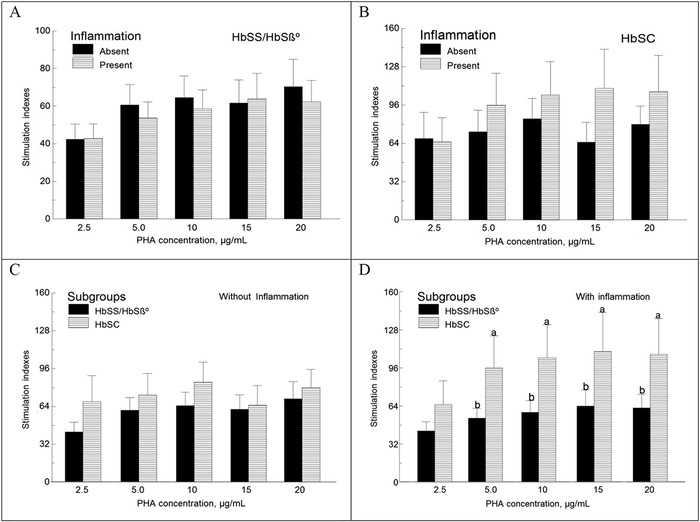
**Effect of inflammation on lymphocyte proliferation in children with the HbSS/HbSβ° genotypes and those with the HbSC genotype. Mononuclear cells were incubated with and without phytohemagglutinin (PHA) for 48 hours before being pulsed with ^3^H-thymidine for 24 hours. Results are expressed as stimulation indexes or the ratio of the amount of radioactivity incorporated into DNA by cells incubated with PHA over baseline. In views A and B, sample sizes are 31 vs 24 and 13 vs 8 for the HbSS/HbSβ° and HbSC subgroups, respectively. Values are means ± standard error of the mean. In the presence of inflammation, the mean stimulation indexes of children with the HbSC genotype were higher than those of children with the HbSS/HbSβ° genotypes (a>b; *P*<0.05).**

Inflammation did not significantly alter lymphocyte proliferation in children with the HbSS/HbSβ° genotypes (Figure 3A). However, inflammation had the opposite effect in the subgroup of children with the HbSC genotype (Figure 3B). In children without inflammation, lymphocyte proliferation was not different among the children with different Hb genotypes (Figure 3C), but in children with inflammation, lymphocyte proliferative responses to PHA concentrations were lower in children with the HbSS/HbSβ° genotype vs those with the HbSC genotype (*P*<0.05) (Figure 3D).

**Figure 4.**
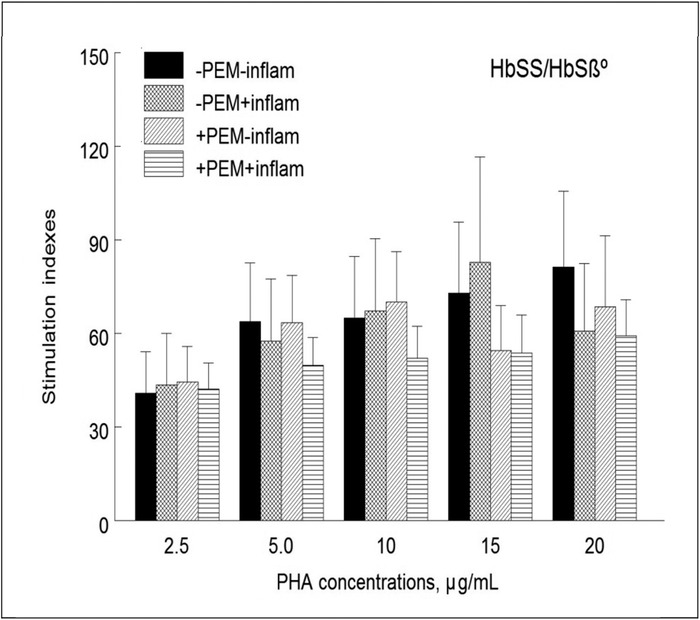
**Lymphocyte proliferation in the subgroup of children with the HbSS/HbSβ° genotypes as a function of undernutrition (protein-energy malnutrition [PEM]) without and with inflammation (inflam). Mononuclear cells were incubated with and without phytohemagglutinin (PHA) before being pulsed with ^3^H-thymidine for 24 hours as explained in the legend for Figure 1. Results are expressed as stimulation indexes. The 4 subgroups are as follows: no PEM or inflammation (n=13); no PEM, inflammation (n=8); PEM, no inflammation (n=14); PEM and inflammation (n=20). Values are means ± standard error of the mean. For each PHA concentration, analysis of variance did not detect significant differences among the 4 subgroups of children.**

We also analyzed lymphocyte proliferation in children with the HbSS/HbSβ° genotypes as a function of both undernutrition and inflammation. Four subgroups were defined: (1) without undernutrition or inflammation, (2) without undernutrition but with inflammation, (3) with undernutrition but without inflammation, and (4) with both undernutrition and inflammation. Although the differences did not reach statistical significance, the mean stimulation indexes of cells treated with PHA concentrations between 5 and 20 μg/mL from children with both undernutrition and inflammation were 20% to 27% lower than those of children without either problem (Figure 4).

We did not analyze lymphocyte proliferation as a function of undernutrition and inflammation for children with the HbSC genotype because too few children met the criteria for the 4 subgroups defined above.

### Interleukin-2 Concentration as a Function of Undernutrition and Inflammation

In the subgroup of children with the HbSS/HbSβ° genotypes, undernutrition did not significantly affect mean IL-2 activity (Table 2). However, in the same children, undernutrition reduced the medians of IL-2 activity in 2 of the 4 cultures by 25.9% to 44.4%. Although the changes did not reach statistical significance, undernutrition slightly reduced the means and medians of IL-2 activity in 3 of the 4 cultures from children with the HbSC genotype.

**Table 2.** Effect of Mild to Moderate Protein-Energy Malnutrition (PEM) on Interleukin-2 Activity in Phytohemagglutinin (PHA)-Treated Peripheral Blood Mononuclear Cells as a Function of Hemoglobin Genotype

		Interleukin-2 Activity, IU/mL			
Group		Without PEM		With PEM	
Genotype	PHA, μg/mL	Mean ± SEM	Median	Mean ± SEM	Median
HbSS/HbSβ° subgroup					
	0	1.11 ± 0.34	0.96	1.59 ± 0.33	1.15
	5	5.34 ± 1.09	5.33	5.41 ± 1.09	3.95
	10	4.32 ± 1.07	2.77	5.11 ± 1.21	4.13
	20	7.92 ± 5.49	2.66	6.19 ± 2.52	1.48
HbSC subgroup					
	0	1.44 ± 0.47	0.96	1.00 ± 0.50	0.43
	5	6.21 ± 1.73	4.51	3.78 ± 2.66	2.11
	10	5.09 ± 1.33	3.77	3.53 ± 2.06	2.04
	20	7.57 ± 4.28	4.37	8.47 ± 5.85	8.48

Notes: Data are presented as means ± standard error of the mean. Sample sizes are 13 without PEM vs 20 with PEM and 8 without PEM vs 4 with PEM for the HbSS/HbSβ° and HbSC subgroups, respectively (interleukin-2 activity was not assayed in some of the samples)*.* No significant differences were observed among children with and without PEM or between genotype subgroups.

**Figure 5.**
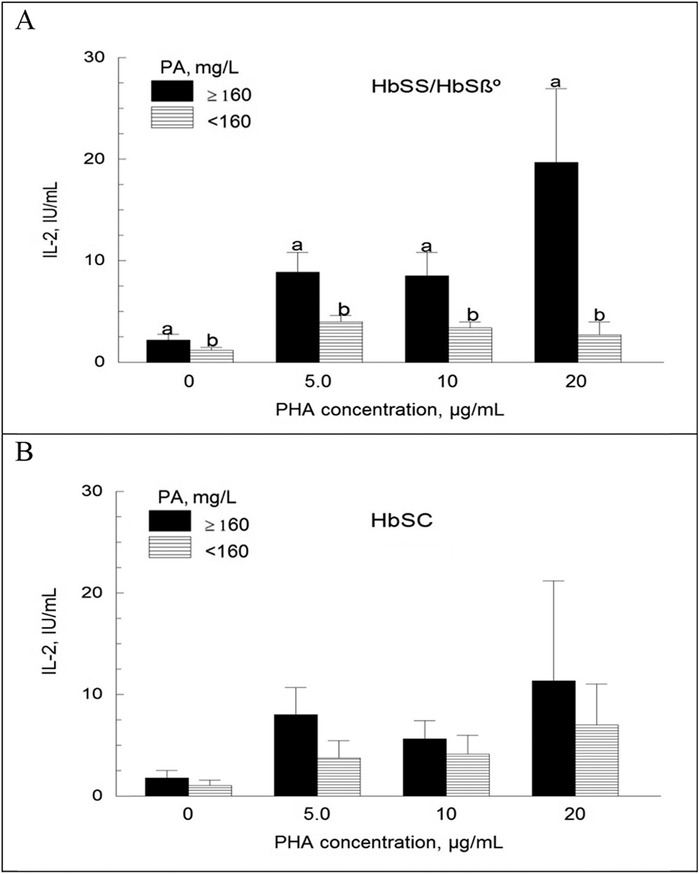
**Interleukin-2 (IL-2) activity in blood mononuclear cell cultures as a function of prealbumin (PA) concentration. Blood mononuclear cells were incubated with and without phytohemagglutinin (PHA) for 48 hours. The supernatants were collected and tested for IL-2 biological activity on IL-2–dependent (CTLL-2) cells. IL-2 was not measured in some of the cultures. Sample sizes are 9 vs 21 and 5 vs 6 in the HbSS/HbSβ° and HbSC subgroups, respectively. Values are means ± standard error of the mean. For each PHA concentration, mean IL-2 biological activity (depicted as bars) with unlike superscript letters are significantly different (a>b; *P*<0.05).**

We also analyzed IL-2 data as a function of individual plasma proteins. For children in both Hb genotype subgroups and for each PHA concentration tested, the mean IL-2 activity of children with PA in the normal range (≥160 mg/L) was higher than that for children with PA <160 mg/L (Figure 5). This difference attained significance (*P*<0.05) only in the HbSS/Hbβ° genotype subgroup. The analysis of other transport proteins (alone or in combination with RBP) did not show such a clear trend (data not shown).

Inflammation did not significantly alter IL-2 activity in cells prepared from children in either of the genotype subgroups (Table 3). Regardless of inflammation status, for each PHA concentration tested, the means for IL-2 activity were not significantly different between the children with the HbSS/Hbβ° genotypes and those with the HbSC genotype.

**Table 3.** Effect of Inflammation on Interleukin-2 Activity in Phytohemagglutinin (PHA)-Treated Peripheral Blood Mononuclear Cells by Hemoglobin Genotype

	Interleukin-2 Activity, IU/mL			
	HbSS/HbSβ° Genotypes Subgroup		HbSC Genotypes Subgroup	
	Without	With	Without	With
	Inflammation	Inflammation	Inflammation	Inflammation
PHA Concentration	(n=19)	(n=13)	(n=8)	(n=3)
0	1.37 ± 0.33	1.64 ± 0.45	1.39 ± 0.50	1.29 ± 0.58
5	5.99 ± 1.18	4.73 ± 1.13	6.37 ± 1.99	3.78 ± 2.66
10	5.23 ± 1.34	4.34 ± 0.90	4.76 ± 1.38	4.12 ± 2.79
20	8.82 ± 4.04	3.74 ± 2.10	6.82 ± 4.46	N/A

Notes: Data are presented as means ± standard error of the mean. No significant difference was observed within and between groups of children with and without inflammation.

N/A, not available (interleukin-2 data were not available for some of the children).

### Correlation Coefficients and Multiple Regression Analysis

We observed positive and significant correlations between lymphocyte proliferative responses to PHA concentrations and both RBP and Hb (*P*<0.05) but not for the other indicators of nutritional status (Table 4). While IL-2 activity positively correlated with PA (*P*<0.05) and weight percentiles (*P*>0.05), it negatively correlated with white blood cell counts (*P*<0.05) and also negatively correlated with Alb (*P*<0.05). IL-2 did not correlate with other indicators of nutritional status or Hb. However, as one would expect, IL-2 negatively correlated with white blood cell counts (PHA 5 and PHA 10; *P*<0.05). Acute phase proteins did not significantly correlate with either lymphocyte proliferative responses to PHA or IL-2 activity (data not shown). Multiple regression analysis did not show any significant association between lymphocyte proliferative responses and/or IL-2 with any one specific indicator of nutritional status (data not shown).

**Table 4.** Correlation Coefficients (r) for Lymphocyte Proliferation (Stimulation Indexes) and Interleukin-2 Activity in Phytohemagglutinin (PHA)-Treated Peripheral Blood Mononuclear Cells and Markers of Nutritional and Hematologic Status in Children With Sickle Cell Disease

	Stimulation Indexes by PHA					Interleukin-2 (IL-2) Activity by			
	Concentration (μg/mL), r					PHA Concentration (μg/mL), r			
Marker	2.5	5	10	15	20	0	5	10	20
Weight percentile	–0.123	–0.116	–0.046	–0.116	0.030	0.0187	0.215	0.254	0.174
Albumin	–0.060	–0.127	–0.150	–0.137	–0.174	–0.348*	–0.169	–0.319*	–0.588*
Prealbumin	0.077	0.035	0.038	–0.027	–0.037	0.383*	0.516*	0.456*	0.542*
Transferrin	–0.022	–0.118	–0.135	–0.132	–0.142	–0.146	–0.233	0.086	–0.145
Retinol binding protein	0.294*	0.372*	0.357*	0.454*	0.440*	–0.184	–0.049	–0.075	0.264
Hemoglobin	0.294*	0.222*	0.246*	0.221	0.293*	0.022	–0.059	0.046	0.210
White blood cell count	–0.160	–0.106	–0.141	–0.087	–0.124	–0.200	–0.268*	–0.260*	–0.095

Notes: Children with the HbSS, HbSβ**°**, and HbSC genotypes were included in the calculations of correlation coefficients. R values (coefficients) with asterisks are different from zero (*P*<0.05). Some *P* values [r] between weight and IL-2 and retinol binding protein and IL-2 that appear as if they should be significant are not significant because of sample size.

## DISCUSSION

The limited available data for patients with SCD suggest that lymphocyte proliferation can be reduced, normal, or occasionally increased compared to subjects without SCD.^30-32^ Differences in lymphocyte proliferation between patients with SCD and subjects without SCD may be related to disease severity (concentrations of sickle [S] and fetal [F] Hb), spleen dysfunction, undernutrition, frequent pain crisis episodes, or infection.^32^

The following are the most important observations from our study.

1. Our results are in accordance with those of Martyres et al who reported that severe growth deficits were uncommon in a study of Canadian children with sickle cell anemia, very likely because of excellent healthcare and disease management.^13^ The low prevalence of severe growth deficits may also suggest that macronutrient intake and utilization were adequate.
2. At the time of recruitment, the prevalence of mild to moderate PEM diagnosed by at least 2 plasma proteins was higher in the subgroup of children with HbSS/HbSβ° disease vs those with the HbSC genotype. The higher prevalence of PEM in the subgroup of children with HbSS/HbSβ° genotypes is in agreement with disease severity as previously reported.^22^
3. Contrary to what we expected, mild to moderate PEM assessed by transport proteins only slightly decreased lymphocyte proliferation in children with the HbSS/HbSβ° genotypes and had no negative effect in those with the HbSC genotype. We speculate that the lack of significant negative effect of PEM on lymphocyte proliferation in these patients is very likely because of the mild form of malnutrition. This speculation is supported by the low number of children who had growth deficits. We must add that lymphocyte proliferative responses to PHA concentrations tended to be reduced in children with the HbSS/HbSβ° genotypes with both PEM and inflammation vs those without PEM ± inflammation. This observation suggests that the health status of children with both problems was worse than that of children without one or both of these problems.
4. Mean lymphocyte proliferative responses to PHA concentrations were reduced in children with RBP ≤20 mg/L and concentrations of Alb, PA, and Tf within normal range vs those with RBP >20 mg/L and the other transport proteins within or below normal range. Our data therefore suggest that, in children with HbSS/HbSβ° disease, mild to moderate malnutrition assessed by RBP alters (reduces) lymphocyte proliferation more so than malnutrition assessed by PA and Tf. Both these plasma proteins are also sensitive although not specific indicators of undernutrition. We do not believe that vitamin A status is the main factor in the association between low lymphocyte proliferative responses to PHA concentration and low RBP level because of the expected low prevalence of vitamin A deficiency in our patients as we previously reported.^19^
5. In parallel to the poorer nutritional status of children with HbSS/HbSβ° genotypes, their in vitro lymphocyte functions also were poorer than those of children with HbSC.
6. This study showed a positive and significant correlation between IL-2 activity and PA but not with Alb, Tf, RBP, and Hb (Table 4). The lack of correlation between in vitro lymphocyte functions with some of the indicators of nutritional status could be related to the fact that we used normal AB serum which is rich in various nutrients that could lead to in vitro repletion. Another possible explanation is the mild to moderate form of PEM in the study population. In fact, <20% of children had 3 to 4 transport proteins below the normal range, and very few had growth deficits. The slight trend of higher lymphocyte proliferative responses to PHA concentration in children with the HbSC genotype with inflammation vs those without inflammation may suggest in vivo cell activation due to underlying unidentified disorders.

We did not investigate the mechanisms of impaired lymphocyte proliferation in children with the HbSS/HbSβ° genotypes in the current study. However, we can speculate several possibilities: (i) differences in the number of immunocompetent (CD4^+^ and CD8^+^) T lymphocytes between children with and without undernutrition, (ii) impaired gene expression of factors that regulate lymphocyte proliferative responses to mitogens, and (iii) altered ratios of effector to regulatory T cells and/or previous blood transfusion.^33,34^

The strengths of our study are the measurement of 4 transport proteins and 3 acute phase proteins for assessment of malnutrition and inflammation, respectively, and the overall sample size. The limitations of our study are the lack of data on mechanisms of impaired lymphocyte functions, plasma retinol concentrations (because vitamin A status regulates RBP biosynthesis), and age-matched African American children without SCD. Another limitation is the small sample size of children with Alb and Tf below the normal range.

## CONCLUSION

Our study suggests that regardless of blood concentrations of Alb, Tf, and PA, children with HbSS/HbSβ° genotypes who had RBP ≤20 mg/L had poorer lymphocyte proliferative responses to PHA concentrations than children with levels >20 mg/L. Inflammation was a confounding factor in altered lymphocyte proliferation in children with HbSS/HbSβ° genotypes. Undernutrition did not reduce lymphocyte proliferation in children with the HbSC genotype. Lymphocyte proliferation was lower in the subgroup of children with the HbSS/HbSβ° genotypes and undernutrition than in the children with the HbSC genotype*.* PA concentration below normal range was associated with reduced mean biological activity of IL-2. The association between mild malnutrition and reduced in vitro lymphocyte function requires further investigation in children with HbSS and/or HbSβ° disease, especially in countries where drugs such as hydroxyurea are less frequently used for the treatment of SCD. Dietary supplements may benefit children with the HbSS/HbSβ° genotypes more than those with the HbSC genotype.

## Notes

This article meets the Accreditation Council for Graduate Medical Education and the American Board of Medical Specialties Maintenance of Certification competencies for Patient Care, Medical Knowledge, and Practice-Based Learning and Improvement.
